# A dataset of bird distributions in zoogeographical regions of China

**DOI:** 10.3897/BDJ.10.e93606

**Published:** 2022-12-29

**Authors:** Qianyi Zhang, Jingru Han, Canwei Xia, Anders Pape Møller

**Affiliations:** 1 Ministry of Education Key Laboratory for Biodiversity and Ecological Engineering, College of Life Sciences, Beijing Normal University, Beijing, China Ministry of Education Key Laboratory for Biodiversity and Ecological Engineering, College of Life Sciences, Beijing Normal University Beijing China; 2 Ministry of Education Key Laboratory for Ecology of Tropical Islands, College of Life Sciences, Hainan Normal University, Haikou, China Ministry of Education Key Laboratory for Ecology of Tropical Islands, College of Life Sciences, Hainan Normal University Haikou China; 3 Université Paris-Saclay, CNRS, AgroParisTech, Ecologie Systématique et Evolution, Gif-sur-Yvette, France Université Paris-Saclay, CNRS, AgroParisTech, Ecologie Systématique et Evolution Gif-sur-Yvette France

**Keywords:** birds, China, distribution, residence type

## Abstract

**Background:**

China, the largest country in Asia, has a land area of approximately 9.6 million square kilometres. There are 1481 bird species (following the taxonomy of IOC World Bird List version 12.1) recorded in two zoogeographical realms, seven regions and 19 subregions in the country. From 1955 to 2017, six authoritative monographs were published, which recorded the distribution area for all bird species in China and were widely quoted by research papers and field guides. This massive amount of data could be used to address many hot topics in ornithology, biogeography and ecology. However, rapid changes in the taxonomic status and different schemes of zoogeographical regionalisation in these six monographs provided limits to the utilisation of these valuable data.

**New information:**

By integrating the data from the six monographs, we presented an open-access dataset on the occurrences and residence types of all Chinese bird species in zoogeographical regions over the past 60 years. The taxonomic statuses for these species were determined following the IOC World Bird List version 12.1 and the zoogeographical regions were based on the updated scheme. These data provide valuable information for the research in bird ecology and conservation biology.

## Introduction

Bird distribution data are vitally important for addressing many hot topics in ecology (Powney and Isaac 2015). Combined with knowledge of phylogeny and ecological traits, the distribution data could contribute to identification of biodiversity hotspots and assessment of conservation prioritisation (Loiseau et al. 2020, Jetz et al. 2014, Benedetti et al. 2022). Macroecology and biogeography also require large-scale distribution data to explain spatial patterns of diversity (Davies et al. 2007, Lennon et al. 2001, Komaki 2021). The impact of global climate change on birds has caused widespread discussion and much of this evidence is based on the knowledge of range shifts (Gillings et al. 2015, Hitch and Leberg 2007, Huntley et al. 2006). Although the occurrence data have been growing continuously under wider public participation, the historical data are difficult to supplement (Peterson et al. 2018). Earlier historical literature may have a positive effect on improving the temporal coverage (Narwade et al. 2011).

China, the largest country in Asia, has a land area of approximately 9.6 million square kilometres and a vast maritime territory (Zheng et al. 2015). The interaction of complex topography and climate provides abundant niches for organisms and breeds high biodiversity in this country (He et al. 2017). According to historical literature and bird surveys, 1481 bird species have been documented in China so far, which is equivalent to 13% of the bird species in the world (Gill et al. 2022, Zheng 2017). However, the existing digital accessible dataset could not fill the gap completely in China’s bird distributions. The most prominent portal of diversity data, Global Biodiversity Information Facility (GBIF) has a number of occurrence data of China’s birds, but the tremendously low temporal coverage hinders researchers from revealing the distributional dynamics of each species (Huang et al. 2020). The Birdlife International and Handbook of the Birds of the World also provide digitised distribution maps for birds, but static maps could not show the distributional dynamics. In addition, the bird species diversity would be underestimated, based on the above digital dataset (Ding et al. 2022). For instance, the record of Velvet Scoter, *Melanittafusca*, in Shannxi Province is not included in the distribution map by Birdlife International (Zheng 2017, Ding et al. 2022).

Since 1955, six Chinese ornithology monographs have been published which were widely quoted by research papers and field guides. These authoritative data could provide historic distributions and geographic dynamics of Chinese birds at the scale of zoogeographical regions. The systematic study of the avifauna in China began in the middle 19^th^ century (Swinhoe 1863). After the middle 20^th^ century, ornithologists carried out many bird surveys and published local avifaunas. Cheng Tso-hsin systematically summarised these data in the first and revised editions of The Distribution List of Chinese Birds (Cheng 1955, Cheng 1958, Cheng 1976) and A Synopsis of the Avifauna of China (Cheng 1987), which were the representative works of bird classification and fauna research at that stage. From the 21^st^ century, the vigorous growth of bird watching activities has provided much new data. Three versions of A Checklist on a Classification and Distribution of the Birds of China (Zheng 2005, Zheng 2011, Zheng 2017) reflected this latest progress in bird classification and distribution.

However, rapid changes in taxonomic status and different schemes of zoogeographical regionalisation in the monographs limit the utilisation of these valuable data. Following IOC World Bird List version 12.1, the taxonomic status of nearly 300 bird species in early monographs need to be updated and the scientific names of more than 400 species also need to be updated due to taxonomic changes. In addition, the standards used to divide zoogeographical regions/subregions are not consistent in the six monographs. Besides, the two versions of The Distribution List of Chinese Birds (Cheng 1955, Cheng 1958, Cheng 1976) written in Chinese were published fifty years ago and are not easily accessible now. All these factors make it difficult to digitise these data. The present project aims to sort out the data from the six monographs under unified standards and provide an open-access dataset about Chinese bird occurrences in zoogeographical regions that could be used in further research to better understand the bird diversity in this country.

## General description

### Purpose

There are six authoritative bird monographs on the occurrence of bird and residence types in China that were published from 1955 to 2017. Rapid changes in taxonomic status and different schemes of zoogeographical regionalisation limit the utilisation of these valuable data. The objective of this study will present a digitalised dataset on bird distributions in zoogeographical regions of China over the past 60 years under unified standards.

## Sampling methods

### Sampling description

The dataset, compiled from six fauna books published from 1955 to 2017 (Cheng 1955, Cheng 1958, Cheng 1976, Cheng 1987, Zheng 2005, Zheng 2011, Zheng 2017), indicates the distributions and residence status of birds in China.

We follow the taxonomy and nomenclature in the IOC World Bird List version 12.1 which is an up-to-date evolutionary classification of world birds constructed by the international community of ornithologists (Gill et al. 2022) and adopt the following strategies to normalise the data: 1) The scientific name would be revised directly if the taxonomy change does not affect its species rank, for example, the scientific name of Swinhoe's Storm Petrel is *Oceanodromamonorhis* in all six monographs, but is changed to *Hydrobatesmonorhis* as the previous genus *Oceanodroma* is paraphyletic (Penhallurick and Wink 2004); 2) The species would be assumed as a non-detection in the time periods before it was discovered, for example, *Stachyrisnonggangensis* is a new species described in 2008 (Zhou and Jiang 2008), which is recorded as a non-detection in the four monographs published before 2008; 3) If a taxon is treated as a synonym of another species, its distribution area would be lumped into the respective species, for example, the species *Caprimulguscentralasicus* is now regarded as a synonym of *Caprimulguseuropaeus* (Schweizer et al. 2020), so the distribution data of *Caprimulguscentralasicus* are merged into *Caprimulguseuropaeus*; 4) If a taxon were regarded as a subspecies previously, but is given a species rank now, its distribution area was adjusted according to the origional document at subspecies level, for example, common blackbird is split into three species, *Turdusmandarinus*, *Turdusmaximus* and *Turdusmerula* (Nylander et al. 2008), the distribution area is adjusted according to the respective subspecies.

**Figure 1.**
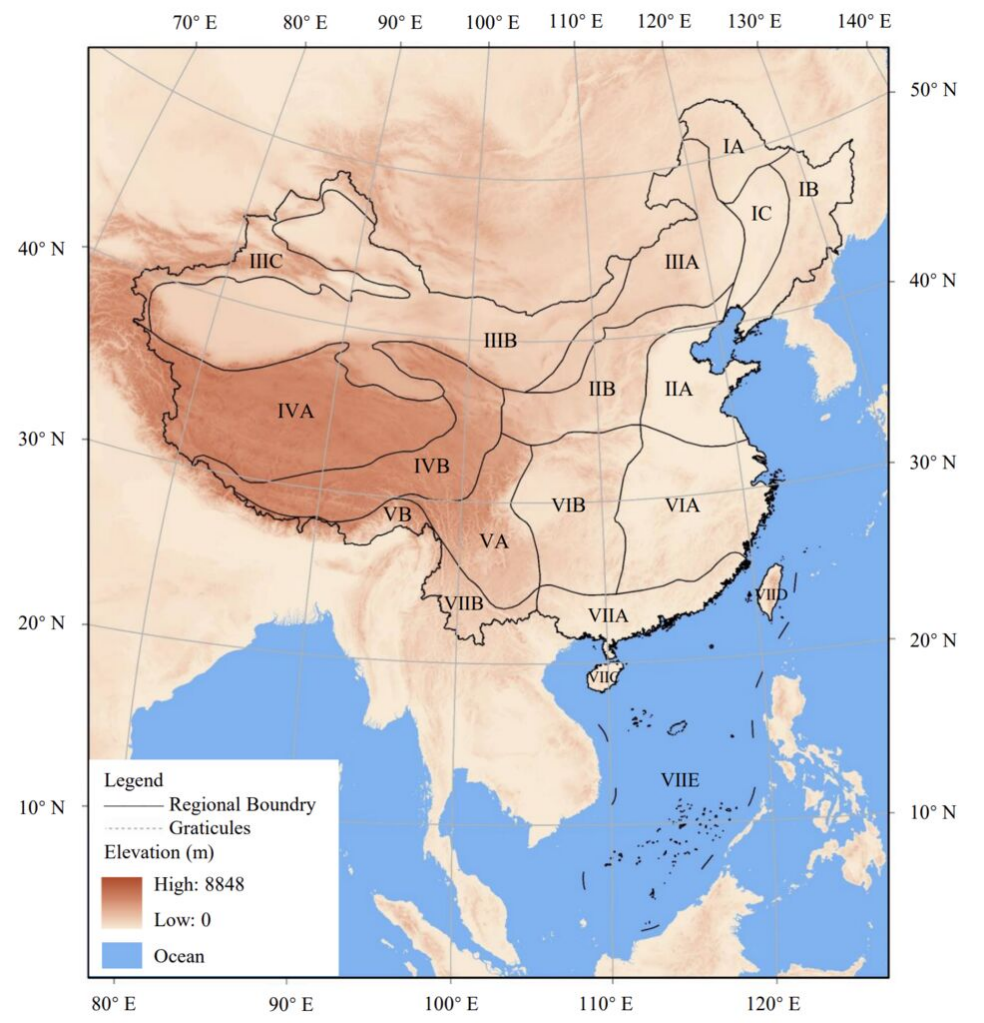
The division of zoogeographical regions of China (Note: Only the land area of each subregion is marked in this figure. Subregion ⅦC includes Hainan Island and adjacent islands, Subregion ⅦD includes Taiwan Island and adjacent islands and Subregion ⅦE includes the islands in the South China Sea).

The first division scheme of China zoogeographical regions was initiated in 1959 and utilised by Cheng in his two monographs (Cheng 1976, Cheng 1987). Then, the scheme was revised twice in 1978 and 1999 (Zhang 1999) and Zheng followed these revisions in his three monographs (Zheng 2005, Zheng 2011, Zheng 2017). The main changes were that: a) the Songliao Plain Subregion, the Himalaya Subregion and the South China Sea Islands Subregion were added so that the total number of subregions increased from 16 to 19; b) the border between the South-western Region and the Qinghai-Xizang Region was adjusted; c) Altay Prefecture was regarded as a part of the West-desert Subregion instead of the Da Hinggan Mountain Subregion. Thus, we follow the zoogeographical regionalisation of China adapted by Zheng (2017) and show the map in Fig. 1 (Zhang 1999). If Cheng was unsure of a distribution, it was noted with a question mark in his monographs (Cheng 1955, Cheng 1958, Cheng 1976, Cheng 1987). These questionable areas were not included in our dataset. For the species distribution boundary close to the boundary between zoogeographical subregions, the data are entered by carefully comparing the distribution maps/textual description of the administrative area with the boundary of the zoogeographical regions (Fig. 1). The data from Zheng's three monographs (Zheng 2005, Zheng 2011, Zheng 2017) are entered according to the records of zoogeographical region codes and provincial administrative units.

For each species, the status in each subregion is indicated in the dataset. The residence types are divided into five categories: resident (R, the birds that live in the subregion all year round and do not migrate in the spring and autumn), summer visitor (S, the birds that come to the subregion for breeding in spring and leave in autumn), winter visitor (W, the birds that come to the subregion for overwintering and leave in spring), passage migrant (P, the birds that stop off in the subregion during their migration, but do not stay there for a long time) and vagrant visitor (V, the birds that occur in the subregion by deviation of the regular path during their migration or are scarce species in the subregion). If the species occurs in the zoogeographical region with particular residence type, it is recorded as “1” in the cell; otherwise, it is recorded as “0” in the cell. It should be noted that there is more than one residence type in the same subregion for many species, for example, Demoiselle crane, *Grusvirgo*, is both a summer visitor and a passage migrant in the north of China (Zheng 2017).

### Quality control

The six monographs on which our dataset is based are the authoritative data of bird distribution in the corresponding period and the information of species distribution is determined by checking specimens, published papers, monographs and local fauna. Therefore, false positives, meaning a species is reported from sites where it does not actually occur, are thought to be rare. The inconsistency between zoogeographical region codes and provincial administrative units was found in a few species. For example, the administrative unit records of *Garrulaxperspicillatus* included most provinces in South China, but not included Qinghai and Xizang Provinces, the zoogeographical region codes in the monographs included ⅣA and ⅣB (cover a lot of areas of Qinghai Tibet Plateau), but not included ⅥA and ⅥB (cover a lot of areas of South China) (Zheng 2005, Zheng 2011, Zheng 2017). Considering *Garrulaxperspicillatus* is a very common species in South China, ⅣA and ⅣB are obviously typographic mistakes of ⅥA and ⅥB. For these cases, we adjusted zoogeographical region codes according to the records of provincial administrative units and contacted the monograph editor to confirm these adjustments. However, the six monographs are phased summaries of bird distributions rather than reports which are based on the same survey protocol and the sites that researchers could reach to collect or observe birds have been limited thus far. Hence, false negatives, in which a species failed to be reported from the sites where it actually occurs, are inevitable in these six monographs. These errors should be considered or corrected when the dataset is used. There are many analytical tools that can deal with distribution data with false negatives (Altwegg and Nichols 2019, Miller et al. 2019).

## Geographic coverage

### Description

China has a land area of approximately 9.6 million square kilometres with two zoogeographical realms, seven regions and 19 subregions (Fig. 1, Table 1).

## Taxonomic coverage

### Description

This dataset provides the distribution information for the 1481 species of birds recorded in China, which belong to 28 orders and 114 families, following IOC World Bird List version 12.1 (Gill et al. 2022) for nomenclature (Table 2).

## Temporal coverage

### Notes

The six monographs were published from 1955 to 2017. The Distribution List of Chinese Birds (Cheng 1955, Cheng 1958) includes bird records since 1922, which makes the state of temporal coverage shift to an earlier date.

## Usage licence

### Usage licence

Creative Commons Public Domain Waiver (CC-Zero)

## Data resources

### Data package title

A dataset of bird distributions in zoogeographical regions of China

### Number of data sets

1

### Data set 1.

#### Data set name

A dataset of bird distributions in zoogeographical regions of China

#### Description

The dataset (Suppl. material 1) which is collected from six avifauna monographs reflects the bird distributions in China in six different time periods from 1955 to 2017 in zoogeographical regions. The taxonomy and nomenclature follow IOC World Bird List (v. 12.1) (Gill et al. 2022) and the zoogeography follows Zhang (1999). The residence type in each subregion of the total 1481 species is recorded by "0" and "1" as presence/absence in the dataset.

**Data set 1.** 

Column label	Column description
The data source	The monograph from which the data were collected (author, year).
Scientific name	The scientific name of the bird.
English name	The English name of the bird.
Chinese name	The Chinese name of the bird.
IAR	The resident in Da Hinggan Mountain Subregion.
IAS	The summer visitor in Da Hinggan Mountain Subregion.
IAW	The winter visitor in Da Hinggan Mountain Subregion.
IAP	The passage migrant in Da Hinggan Mountain Subregion.
IAV	The vagrant visitor in Da Hinggan Mountain Subregion.
IBR	The resident in Changbai Mountain Subregion.
IBS	The summer visitor in Changbai Mountain Subregion.
IBW	The winter visitor in Changbai Mountain Subregion.
IBP	The passage migrant in Changbai Mountain Subregion.
IBV	The vagrant migrant in Changbai Mountain Subregion.
ICR	The resident in Songliao Plain Subregion.
ICS	The summer visitor in Songliao Plain Subregion.
ICW	The winter visitor in Songliao Plain Subregion.
ICP	The passage migrant in Songliao Plain Subregion.
ICV	The vagrant migrant in Songliao Plain Subregion.
IIAR	The resident in Huang-Huai Plain Subregion.
IIAS	The summer visitor in Huang-Huai Plain Subregion.
IIAW	The winter vistor in Huang-Huai Plain Subregion.
IIAP	The passage migrant in Huang-Huai Plain Subregion.
IIAV	The vagrant migrant in Huang-Huai Plain Subregion.
IIBR	The resident in Loess Plateau Subregion.
IIBS	The summer visitor in Loess Plateau Subregion.
IIBW	The winter visitor in Loess Plateau Subregion.
IIBP	The passage migrant in Loess Plateau Subregion.
IIBV	The vagrant migrant in Loess Plateau Subregion.
IIIAR	The resident in East-Meadow Subregion.
IIIAS	The summer visitor in East-Meadow Subregion.
IIIAW	The winter visitor in East-Meadow Subregion.
IIIAP	The passage migrant in East-Meadow Subregion.
IIIAV	The vagrant migrant in East-Meadow Subregion.
IIIBR	The resident in West-desert Subregion.
IIIBS	The summer visitor in West-desert Subregion.
IIIBW	The winter visitor in West-desert Subregion.
IIIBP	The passage migrant in West-desert Subregion.
IIIBV	The vagrant migrant in West-desert Subregion.
IIICR	The resident in Tianshan Hilly Subregion.
IIICS	The summer visitor in Tianshan Hilly Subregion.
IIICW	The winter visitor in Tianshan Hilly Subregion.
IIICP	The passage migrant in Tianshan Hilly Subregion.
IIICV	The vagrant migrant in Tianshan Hilly Subregion.
IVAR	The resident in Qiantang Plateau Subregion.
IVAS	The summer vistitor in Qiantang Plateau Subregion.
IVAW	The winter visitor in Qiantang Plateau Subregion.
IVAP	The passage migrant in Qiantang Plateau Subregion.
IVAV	The vagrant migrant in Qiantang Plateau Subregion.
IVBR	The resident in Qinghai-Zangnan Subregion.
IVBS	The summer visitor in Qinghai-Zangnan Subregion.
IVBW	The winter visitor in Qinghai-Zangnan Subregion.
IVBP	The passage migrant in Qinghai-Zangnan Subregion.
IVBV	The vagrant migrant in Qinghai-Zangnan Subregion.
VAR	The resident in South-West Mountains Subregion.
VAS	The summer visitor in South-West Mountains Subregion.
VAW	The winter visitor in South-West Mountains Subregion.
VAP	The passage migrant in South-West Mountains Subregion.
VAV	The vagrant migrant in South-West Mountains Subregion.
VBR	The resident in Himalaya Subregion.
VBS	The summer visitor in Himalaya Subregion.
VBW	The winter visitor in Himalaya Subregion.
VBP	The passage migrant in Himalaya Subregion.
VBV	The vagrant migrant in Himalaya Subregion.
VIAR	The resident in Eastern Hillock-Plain Subregion.
VIAS	The summer visitor in Eastern Hillock-Plain Subregion.
VIAW	The winter visitor in Eastern Hillock-Plain Subregion.
VIAP	The passage migrant in Eastern Hillock-Plain Subregion.
VIAV	The vagrant migrant in Eastern Hillock-Plain Subregion.
VIBR	The resident in Western Mountain-Plateau Subregion.
VIBS	The summer visitor in Western Mountain-Plateau Subregion.
VIBW	The winter visitor in Western Mountain-Plateau Subregion.
VIBP	The passage migrant in Western Mountain-Plateau Subregion.
VIBV	The vagrant migrant in Western Mountain-Plateau Subregion.
VIIAR	The resident in Min-Guang Coastal Subregion.
VIIAS	The summer visitor in Min-Guang Coastal Subregion.
VIIAW	The winter visitor in Min-Guang Coastal Subregion.
VIIAP	The passage migrant in Min-Guang Coastal Subregion.
VIIAV	The vagrant migrant in Min-Guang Coastal Subregion.
VIIBR	The resident in Diannan Hilly Subregion.
VIIBS	The summer visitor in Diannan Hilly Subregion.
VIIBW	The winter visitor in Diannan Hilly Subregion.
VIIBP	The passage migrant in Diannan Hilly Subregion.
VIIBV	The vagrant migrant in Diannan Hilly Subregion.
VIICR	The resident in Hainan Island Subregion.
VIICS	The summer visitor in Hainan Island Subregion.
VIICW	The winter visitor in Hainan Island Subregion.
VIICP	The passage migrant in Hainan Island Subregion.
VIICV	The vagrant migrant in Hainan Island Subregion.
VIIDR	The resident in Taiwan Subregion.
VIIDS	The summer visitor in Taiwan Subregion.
VIIDW	The winter visitor in Taiwan Subregion.
VIIDP	The passage migrant in Taiwan Subregion.
VIIDV	The vagrant migrant in Taiwan Subregion.
VIIER	The resident in South China Sea Islands Subregion.
VIIES	The summer visitor in South China Sea Islands Subregion.
VIIEW	The winter visitor in South China Sea Islands Subregion.
VIIEP	The passage migrant in South China Sea Islands Subregion.
VIIEV	The vagrant migrant in South China Sea Islands Subregion.
Note	Notes of the status and taxonomic changes.

## Supplementary Material

5CB8E7C3-BFA6-51C1-A668-7D60B6ECE87C 10.3897/BDJ.10.e93606.suppl1 Supplementary material 1 A dataset of bird distributions in zoogeographical regions of China Data type occurrences Brief description The dataset which is collected from six avifauna monographs reflects the bird distributions in China in six different time periods from 1955 to 2017 in zoogeographical regions. The taxonomy and nomenclature follow the IOC World Bird List (v. 12.1) (Gill et al. 2022) and the zoogeography follows Zhang (1999). The residence type in each subregion of the total 1481 species is recorded by "0" and "1" as presence/absence in the dataset. File: oo_782125.txt https://binary.pensoft.net/file/782125 Qianyi Zhang, Jingru Han, Canwei Xia, Anders Pape Møller

## Figures and Tables

**Table 1.** The division of zoogeographical regions of China (Note: Only the land area of each subregion is calculated in this table. Subregion ⅦC includes Hainan Island and adjacent islands and the centre of Hainan Island is recorded as the geometric centre of this subregion. Subregion ⅦD includes Taiwan Island and adjacent islands and the centre of Taiwan Islands is recorded as the geometric centre of this subregion. Subregion ⅦE includes the islands in the South China Sea and Huangyan Dao is regarded as the geometric centre of this subregion).

Realm	Zoogeographical region	Zoogeographical subregion	Ecogeographical Fauna Group	Area	Geometric centre
Palearctic Realm	Ⅰ. North-eastern Region	IA. Da Hinggan Mountain Subregion	Boreal forest fauna	2.5×10^5^ km^2^	50.59°N, 124.15°E
		ⅠB. Changbai Mountain Subregion	Temperate forest, forest-steppe and farmland fauna	4.1×10^5^ km^2^	45.15°N, 125.00°E
		ⅠC. Songliao Plain Subregion	Temperate forest, forest-steppe and farmland fauna	2.5×10^5^ km^2^	44.59°N, 128.70°E
	Ⅱ. North China Region	ⅡA. Huang-Huai Plain Subregion	Temperate forest, forest-steppe and farmland fauna	4.1×10^5^ km^2^	36.37°N, 117.16°E
		ⅡB. Loess Plateau Subregion	Temperate forest, forest-steppe and farmland fauna	5.7×10^5^ km^2^	37.43°N, 110.71°E
	Ⅲ. Mongo-Xinjiang Region	ⅢA. East-Meadow Subregion	Temperate steppe fauna	7.0×10^5^ km^2^	44.43°N, 117.12°E
		ⅢB. West-desert Subregion	Temperate desert and semi-desert fauna	1.9×10^6^ km^2^	40.63°N, 92.18°E
		ⅢC. Tianshan Hilly Subregion	Alpine forest-steppe, meadow steppe and cold desert fauna	4.1×10^5^ km^2^	43.34°N, 83.91°E
	Ⅳ. Qinghai-Xizang Region	ⅣA. Qiantang Plateau Subregion	Alpine forest-steppe, meadow steppe and cold desert fauna	1.2×10^6^ km^2^	34.03°N, 87.51°E
		ⅣB. Qinghai-Zangnan Subregion	Alpine forest-steppe, meadow steppe and cold desert fauna	8.2×10^5^ km^2^	32.86°N, 95.37°E
Oriental Realm	Ⅴ. South-western Region	ⅤA. South-West Mountains Subregion	Subtropical forest, scrub, grassland and farmland fauna	5.2×10^5^ km^2^	28.45°N, 102.12°E
		ⅤB. Himalaya Subregion	Subtropical forest, scrub, grassland and farmland fauna	1.3×10^5^ km^2^	28.39°N, 93.65°E
	Ⅵ. Mid-China Region	ⅥA. Eastern Hillock-Plain Subregion	Subtropical forest, scrub, grassland and farmland fauna	8.6×10^5^ km^2^	29.21°N, 115.95°E
		ⅥB. Western Mountain-Plateau Subregion	Subtropical forest, scrub, grassland and farmland fauna	7.1×10^5^ km^2^	29.71°N, 108.33°E
	Ⅶ. South China Region	ⅦA. Min-Guang Coastal Subregion	Tropical forest, scrub, grassland and farmland fauna	3.7×10^5^ km^2^	23.43°N, 111.75°E
		ⅦB. Diannan Hilly Subregion	Tropical forest, scrub, grassland and farmland fauna	1.9×10^5^ km^2^	23.88°N, 100.77°E
		ⅦC. Hainan Island Subregion	Tropical forest, scrub, grassland and farmland fauna	3.5×10^4^ km^2^	19.19°N, 109.74°E
		ⅦD. Taiwan Subregion	Tropical forest, scrub, grassland and farmland fauna	3.6×10^4^ km^2^	23.75°N, 120.97°E
		ⅦE. South China Sea Islands Subregion	Tropical forest, scrub, grassland and farmland fauna	2.0×10^2^ km^2^	15.11°N, 117.46°E

**Table 2.** The taxa included in The Dataset on the Birds Distribution in China over the past 60 years

Rank	Scientific name	Number of families	Number of species
Order	Anseriformes	1	55
Order	Galliformes	1	64
Order	Caprimulgiformes	1	6
Order	Podargiformes	1	1
Order	Apodiformes	2	16
Order	Otidiformes	1	3
Order	Pterocliformes	1	3
Order	Columbiformes	1	31
Order	Cuculiformes	1	20
Order	Gruiformes	2	29
Order	Podicipediformes	1	5
Order	Phoenicopteriformes	1	1
Order	Charadriiformes	13	135
Order	Phaethontiformes	1	3
Order	Gaviiformes	1	4
Order	Procellariiformes	4	16
Order	Ciconiiformes	1	8
Order	Suliformes	3	11
Order	Pelecaniformes	3	35
Order	Accipitriformes	2	55
Order	Strigiformes	2	33
Order	Trogoniformes	1	3
Order	Bucerotiformes	2	6
Order	Coraciiformes	3	23
Order	Piciformes	3	43
Order	Falconiformes	1	12
Order	Psittaciformes	1	10
Order	Passeriformes	59	850
